# Pathological and perioperative outcomes of extracorporeal *versus* intracorporeal anastomosis in laparoscopic transverse colon cancer resection: retrospective multicentre study

**DOI:** 10.1093/bjsopen/zrad045

**Published:** 2023-05-10

**Authors:** Hao Zhong, Zhenghao Cai, Junyang Lu, Yingchi Yang, Qing Xu, Nan Wang, Liang He, Xiyue Hu, Abraham Fingerhut, Minhua Zheng, Aiguo Lu, Zheng Liu, Yi Xiao, Bo Feng

**Affiliations:** Department of General Surgery, Ruijin Hospital, Shanghai Jiao Tong University School of Medicine, Shanghai, China; Department of General Surgery, Ruijin Hospital, Shanghai Jiao Tong University School of Medicine, Shanghai, China; Department of General Surgery, Peking Union Medical College Hospital, Beijing, China; Department of General Surgery, Beijing Friendship Hospital, Capital Medical University, Beijing, China; Department of General Surgery, Renji Hospital, Shanghai Jiao Tong University School of Medicine, Shanghai, China; Department of General Surgery, Tangdu Hospital, Shanxi, China; Department of General Surgery, The First Hospital of Jilin University, Jilin, China; Department of Colorectal Surgery, Cancer Hospital Chinese Academy of Medical Sciences, Beijing, China; Section for Surgical Research, Department of Surgery, Medical University of Graz, Graz, Austria; Department of General Surgery, Ruijin Hospital, Shanghai Jiao Tong University School of Medicine, Shanghai, China; Department of General Surgery, Ruijin Hospital, Shanghai Jiao Tong University School of Medicine, Shanghai, China; Department of Colorectal Surgery, Cancer Hospital Chinese Academy of Medical Sciences, Beijing, China; Department of General Surgery, Peking Union Medical College Hospital, Beijing, China; Department of General Surgery, Ruijin Hospital, Shanghai Jiao Tong University School of Medicine, Shanghai, China

## Abstract

**Background:**

The aim of this study was to compare the pathological and perioperative outcomes of extracorporeal *versus* intracorporeal anastomosis after laparoscopic transverse colon cancer resection.

**Methods:**

In this retrospective study, patients from seven institutions in China who underwent laparoscopic resection of transverse colon cancer between 2019 and 2021 were selected and included. Either extended right hemicolectomy or transverse colectomy/extended left hemicolectomy was performed. The clinical characteristics and the pathological and perioperative outcomes were compared between patients undergoing extracorporeal or intracorporeal anastomosis. Resection margin lengths were measured on formalin-fixed specimens and an inadequate margin was defined as less than 4.2 cm between the division and the tumour. The outcome of interest was the prevalence of specimens with an inadequate margin. Length of incision, bowel function recovery, hospital stay, early postoperative pain (first day after surgery), 30-day complications, and nodal harvest were investigated as secondary outcomes.

**Results:**

Of 411 patients treated during the study interval, 370 patients with transverse colon cancer were included (23.2 per cent treated with intracorporeal anastomosis and 76.8 per cent treated with extracorporeal anastomosis). The prevalence of specimens with inadequate margins was lower in the intracorporeal anastomosis group compared with the extracorporeal anastomosis group in patients undergoing extended right hemicolectomy (*P* = 0.045) and in patients undergoing transverse colectomy/extended left hemicolectomy (*P* = 0.030). In multivariate analysis, extracorporeal anastomosis (OR 2.94 (95 per cent c.i. 1.33 to 6.49), *P* = 0.008) and transverse colectomy/extended left hemicolectomy (OR 1.75 (95 per cent c.i. 1.03 to 2.96), *P* = 0.038) were independent risk factors for specimens with an inadequate margin. Intracorporeal anastomosis was associated with a shorter incision length (*P* < 0.001), an earlier recovery of bowel function (*P* = 0.035), a shorter postoperative hospital stay (*P* = 0.042), less early postoperative pain (*P* < 0.001), a longer specimen length (*P* = 0.042), a longer resection margin (*P* = 0.007), and a greater lymph node harvest (*P* = 0.036). There was no statistically significant difference in 30-day complications.

**Conclusion:**

Patients with transverse colon cancer have better perioperative outcomes, fewer margins of less than 4.2 cm, and larger lymph node harvests when the anastomosis is performed intracorporeally. Further studies are needed to confirm these findings.

**Registration number:**

NCT05061199 (www.clinicaltrials.gov).

## Teaser

In this study, we compared two approaches of anastomosis after laparoscopic resection of transverse colon cancer. We concluded that laparoscopic resection of transverse colon cancer with intracorporeal anastomosis showed superiority in perioperative outcomes and pathological specimen quality. Patients undergoing intracorporeal anastomosis might have more adequate resection margins and a greater lymph node yield.

## Introduction

Colorectal cancer (CRC) is the third most common cancer worldwide^1^. Several RCTs have documented the benefits of laparoscopic resection of CRC in terms of morbidity and hospital stay compared with open surgery^2–5^. With the development of laparoscopic colectomy, intracorporeal anastomosis (ICA) was introduced as a natural alternative to extracorporeal anastomosis (ECA) to restore continuity of the colon after resection^6^. Compared with ECA, ICA leads to a smaller incision (no need to extract both limbs of the colon at the same time for the anastomosis), and less traction on colonic segments, thought to be associated with earlier return of bowel function, shorter hospital stay, and decreased morbidity in both laparoscopic right and left hemicolectomy^7–10^.

Clear resection margins and adequate lymphadenectomy are the key features of surgical oncology for radical resection, and in relation to these elements the oncological performance of ICA has been investigated. Several studies have found that laparoscopic right hemicolectomy with ICA leads to longer surgical specimens with uninvolved resection margins and also higher lymph node harvests^11–14^, although other studies found that the difference in margins could only be observed in tumours located in the transverse colon and hepatic flexure, not in tumours located in the caecum and ascending colon^13^.

A transverse colon cancer (TCC) is defined as a tumour located between the hepatic and splenic flexures; overall, TCC accounts for 10 per cent of all colon cancer^15^. Due to the complexity of anatomy, the diversity of resection extent (extended right hemicolectomy (ERHC), extended left hemicolectomy (ELHC), or transverse colectomy (TC)), and the lack of high-quality evidence from clinical trials^16–18^, the technique for laparoscopic TCC surgery is not standardized, in particular with respect to ICA or ECA^19^.

Thus, in this study, the primary aim was to investigate TCC patients undergoing surgical resection with extracorporeal and intracorporeal anastomoses in relation to pathological findings and perioperative outcomes.

## Methods

The study protocol was approved by the Ruijin Hospital Ethics Committee (2021–353) and was registered on clinicaltrials.gov (NCT05061199). The requirement of informed consent was waived due to the retrospective design of the study.

### Patients

Patients from seven participating centres who underwent laparoscopic resection of TCC between July 2019 and July 2021 were reviewed for inclusion in the study. The participating centres were Shanghai Ruijin Hospital, Peking Union Medical College Hospital, Shanghai Renji Hospital, The First Hospital of Jilin University, Beijing Friendship Hospital, Tangdu Hospital, and Cancer Hospital Chinese Academy of Medical Sciences. Centres were required to document having performed at least 5 colectomies with ICA and 15 with ECA for TCC to participate in the study. All surgeries were performed by a single operation team in each participating centre. The surgeons’ experience is shown in *Table S1*. The inclusion criteria were: pathologically confirmed adenocarcinoma of the colon; tumour located in the transverse colon including the hepatic or splenic flexure (based on operative records); treated with one of the following procedures: ERHC, TC, or ELHC; use of laparoscopic surgery (laparoscopy-assisted surgery or complete laparoscopic surgery); and a radical resection (R0). Exclusion criteria were: synchronous multiple CRC tumour located in the ascending colon close to the hepatic flexure or the descending colon close to the splenic flexure; past surgical history of proctocolectomy; robot-assisted surgery; palliative resection (non-R0 resection) or palliative bypass; emergency procedure; and impossible to determine how the anastomosis was performed from patient records. Patients were divided into two groups according to how anastomosis was performed, ICA or ECA.

### Surgical technique

The mobilization of the intestines and the lymph node dissection were performed following the complete mesocolon excision (CME) principle, in accordance with the national guideline for laparoscopic radical resection of CRC (2018 edition)^20^. The extent of ERHC or TC/ELHC was determined by the vessels disconnected during the surgical procedure. In the ERHC procedure, ileocolic vessels, right colic vessels, and the root of middle colic vessels were disconnected. In the TC/ELHC procedure, the root of middle colic vessels with or without left colic vessels was disconnected. For ICA, a side-to-side isoperistaltic or antiperistaltic anastomosis was performed, using an endoscopic 60-mm linear stapler. The enterotomy was closed using a single-layer continuous barbed suture after isoperistaltic anastomosis. A linear stapler was used to close the enterotomy after antiperistaltic anastomosis. Then the specimen was extracted via median periumbilical or Pfannenstiel incision (3–5 cm). For ECA, the mobilized colon was exteriorized through a 6–10 cm median periumbilical, right paramedian, or left paramedian incision, according to tumour location. Then, a hand-sewn or mechanical anastomosis was performed. All centres used the same standard perioperative care, based on guidelines for laparoscopic radical CRC surgery^21^.

### Data collection

Demographics (sex, age, BMI), perioperative outcomes (estimated blood loss, operative time, incision length), pathological results (length of resection margins, lymph node harvest), and postoperative complications were retrospectively analysed. All data were extracted from the electronic hospital record system with the permission of the Ethics Committee of each participating centre (raw data are available at https://crc.c-increase.com).

### Outcomes of interest

The primary outcome of interest was the prevalence of specimens with an inadequate margin (less than 4.2 cm). Of note, the length of resection margins was measured on a formalin-fixed specimen in all seven centres. Given an optimal margin of 5 cm, this cut-off was set considering that the resection margin of colon cancer presents with a 15.3 per cent shrinkage after formalin fixation^22^ (5.0 cm × (1–0.153) = 4.2 cm).

The secondary outcomes included other pathological results (nodal harvest, pT stage, pN stage, AJCC stage, specimen length) and perioperative indicators (operative time, blood loss, bowel function recovery, rescue analgesic usage, hospital stay).

Postoperative pain was measured on day 1 (POD1) with a visual analogue scale (VAS) ranging from 0 to 10 (0 = no pain, 10 = maximal pain). Non-steroidal anti-inflammatory drugs were administered as the rescue analgesic when the VAS was greater than 5. Postoperative complications within 30 days were assessed according to the Clavien–Dindo classification^23^ and complications greater than or equal to grade 2 were recorded and analysed.

### Statistical analysis

Statistical analyses were performed using SPSS^®^ (IBM, Armonk, NY, USA; 22.0 version). The Kolmogorov–Smirnov test was used to evaluate the data distribution. Student’s *t* test was used for continuous variables with a normal distribution. Otherwise, the Mann–Whitney *U* test was used. The chi-squared test or Fisher’s exact probability test was used for categorical variables, as appropriate. Multivariate analysis was performed using binary logistic regression. *P* values less than 0.050 were considered to be statistically significant. All variables with *P* < 0.100 in the univariate analysis were included in the multivariate analysis.

## Results

### Study population

**Fig. 1:**
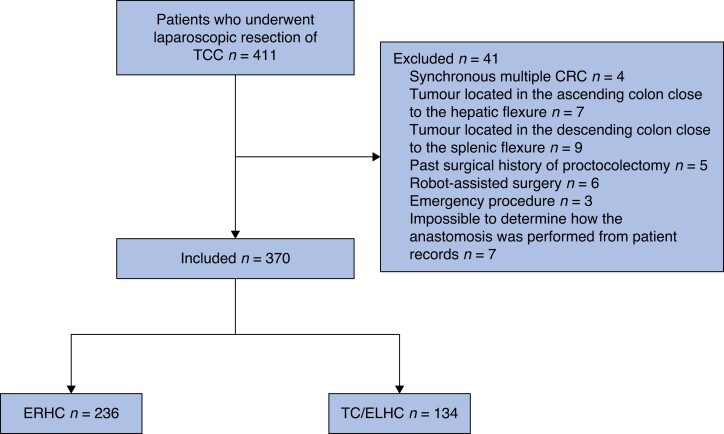
**Study flow chart**TCC, transverse colon cancer; CRC, colorectal cancer; ERHC, extended right hemicolectomy; TC, transverse colectomy; ELHC, extended left hemicolectomy.

Of 411 eligible patients, 370 patients were included in the study (*Fig. 1*). ERHC was performed in 236 patients, of whom 66 and 170 patients underwent ICA and ECA respectively (*Table 1*). TC/ELHC was performed in 134 patients, of whom 20 and 114 patients underwent ICA and ECA respectively (*Table 2*). There were no significant differences between the two groups with regard to baseline characteristics. All cases were completed laparoscopically without conversion to open surgery.

**Table 1:** Patient demographics, pathology, and short-term results in extended right hemicolectomy (*n* = 236)

Variables	ICA group (*n* = 66)	ECA group (*n* = 170)	*P*
**Sex**			0.401
Male	42 (63.6)	98 (57.6)	
Female	24 (36.4)	72 (42.4)	
Age (years), mean(s.d.)	60.3(12.9)	60.9(13.1)	0.777
BMI (kg/m^2^), mean(s.d.)	24.2(3.3)	23.6(3.4)	0.127
**ASA grade**			0.276
I	14 (21.2)	54 (31.8)	
II	47 (71.2)	108 (63.5)	
III	4 (6.1)	8 (4.7)	
Missing	1 (1.5)	0 (0.0)	
**Tumour location**			0.907
Transverse colon	41 (62.1)	107 (62.9)	
Hepatic flexure	25 (37.9)	63 (37.1)	
**Clinical stage**			0.116
I	3 (4.5)	13 (7.6)	
II	21 (31.8)	74 (43.5)	
III	42 (63.6)	83 (48.8)	
Operative time (min), median (range)	150.0 (95.0–260.0)	140.0 (80.0–329.0)	0.039
**Anastomosis technique**			<0.001
Hand sewn	0 (0.0)	6 (3.5)	
End-to-side anastomosis with circular staple	0 (0.0)	80 (47.1)	
Side-to-side isoperistaltic anastomosis	35 (53.0)	34 (20.0)	
Side-to-side antiperistaltic anastomosis	31 (47.0)	49 (28.8)	
Missing	0 (0.0)	1 (0.6)	
Estimated blood loss (ml), median (range)	20.0 (10.0–200.0)	20.0 (5.0–250.0)	0.237
Incision length (cm), median (range)	4.5 (2.5–6.0)	7.5 (4.0–10.0)	<0.001
**Extraction site**			<0.001
Median periumbilical	28 (42.4)	77 (45.3)	
Right paramedian	0 (0.0)	93 (54.7)	
Pfannenstiel incision	38 (57.6)	0 (0.0)	
Pain (VAS), median (range)	1.5 (1.0–4.0)	3.5 (1.0–8.0)	<0.001
Rescue analgesic usage	6 (9.1)	35 (20.6)	0.036
Recovery of bowel function (days), median (range)	2.0 (2.0–3.0)	3.0 (2.0–4.0)	0.035
Postoperative hospital stay (days), median (range)	6.0 (3.0–17.0)	7.0 (4.0–39.0)	0.042
**Postoperative complications**	3 (4.5)	12 (7.1)	0.568
Wound infection	0 (0.0)	2 (1.2)	
Anastomotic leakage	1 (1.5)	2 (1.2)	
Anastomotic bleeding	0 (0.0)	1 (0.6)	
Ileus	1 (1.5)	1 (0.6)	
SSI	0 (0.0)	1 (0.6)	
Pneumonia	0 (0.0)	2 (1.2)	
Urinary tract infection	1 (1.5)	0 (0.0)	
Gastroparesis	0 (0.0)	1 (0.6)	
Others	0 (0.0)	2 (1.2)	
**Clavien–Dindo grade**			0.188
0–I	63 (95.5)	158 (92.9)	
II	2 (3.0)	12 (7.1)	
III	1 (1.5)	0 (0.0)	
Maximum tumour diameter (mm), median (range)	43.0 (15.0–100.0)	46.0 (8.0–140.0)	0.314
**pT stage**			0.497
1	6 (9.1)	8 (4.7)	
2	4 (6.1)	11 (6.5)	
3	32 (48.5)	95 (55.9)	
4	24 (36.4)	56 (32.9)	
**pN stage**			0.344
0	38 (57.6)	114 (67.1)	
1	18 (27.3)	39 (22.9)	
2	10 (15.2)	17 (10.0)	
**AJCC stage**			0.368
1	7 (10.6)	18 (10.6)	
2	31 (47.0)	96 (56.5)	
3	28 (42.4)	56 (32.9)	
Specimen length (cm), median (range)	28.3 (13.0–68.0)	24.0 (4.5–62.0)	0.042
**Margin (cm), median (range)**			
Proximal margin	17.8 (5.0–57.0)	15.8 (3.0–49.0)	0.258
Distal margin	6.0 (1.5–17.0)	5.0 (0.5–19.0)	0.007
Specimen with an inadequate margin	6 (9.1)	34 (20.0)	0.045
**Lymph node, median (range)**		Missing = 1	
Total removed	25.0 (10.0–88.0)	20.0 (2.0–81.0)	0.036
Positive	0.0 (0.0–9.0)	0.0 (0.0–13.0)	0.187

Values are *n* (%) unless otherwise stated. ICA, intracorporeal anastomosis; ECA, extracorporeal anastomosis; VAS, visual analogue scale; SSI, surgical site infection.

**Table 2:** Patient demographics, pathology, and short-term results in transverse colectomy/extended left hemicolectomy (*n* = 134)

Variables	ICA group (*n* = 20)	ECA group (*n* = 114)	*P*
**Sex**			0.028
Male	7 (35.0)	70 (61.4)	
Female	13 (65.0)	44 (38.6)	
Age (years), mean(s.d.)	58.1(11.4)	60.2(12.5)	0.723
BMI (kg/m^2^), mean(s.d.)	22.9(3.4)	24.1(3.2)	0.899
**ASA grade**			0.145
I	1 (5.0)	26 (22.8)	
II	18 (90.0)	82 (71.9)	
III	1 (5.0)	6 (5.3)	
**Tumour location**			0.409
Transverse colon	16 (80.0)	81 (71.1)	
Splenic flexure	4 (20.0)	33 (28.9)	
**Clinical stage**			0.923
I	1 (5.0)	7(6.1)	
II	10 (50.0)	51 (44.7)	
III	9 (45.0)	56 (49.1)	
Operative time (min), median (range)	135.5 (86.0–389.0)	150.0 (55.0–317.0)	0.540
**Anastomosis technique**			<0.001
Hand sewn	0 (0.0)	15 (13.2)	
End-to-side anastomosis with circular staple	0 (0.0)	42 (36.8)	
Side-to-side isoperistaltic anastomosis	7 (35.0)	22 (19.3)	
Side-to-side antiperistaltic anastomosis	13 (65.0)	35 (30.7)	
Estimated blood loss (ml), median (range)	22.5 (10.0–200.0)	20.0 (5.0–300.0)	0.453
Incision length (cm), median (range)	4.5 (2.5–6.5)	7.5 (4.0–11.0)	<0.001
**Extraction site**			<0.001
Median periumbilical incision	6 (30.0)	73 (64.0)	
Left paramedian incision	0 (0.0)	41 (36.0)	
Pfannenstiel incision	14 (70.0)	0 (0.0)	
Pain (VAS), median (range)	2.0 (1.0–4.0)	4.0 (1.0–8.0)	<0.001
Rescue analgesic usage	1 (5.0)	29 (25.4)	0.045
Recovery of bowel function (days), median (range)	2.0 (2.0–3.0)	3.0 (2.0–5.0)	0.034
Postoperative hospital stay (days), median (range)	6.0 (5.0–15.0)	7.0 (4.0–19.0)	0.039
**Postoperative complications**	1 (5.0)	11 (9.6)	0.693
Wound infection	0 (0.0)	2 (1.8)	
Anastomotic leakage	0 (0.0)	1 (0.9)	
Anastomotic bleeding	1 (5.0)	1 (0.9)	
Ileus	0 (0.0)	1 (0.9)	
Pneumonia	0 (0.0)	2 (1.8)	
Urinary tract infection	0 (0.0)	1 (0.9)	
Gastroparesis	0 (0.0)	1 (0.9)	
Others	0 (0.0)	2 (1.8)	
**Clavien–Dindo grade**			0.693
0–I	19 (95.0)	103 (90.4)	
II	1 (5.0)	11 (9.6)	
Maximum tumour diameter (mm), median (range)	42.5 (13.0–65.0)	40.0 (8.0–105.0)	0.742
**pT stage**			0.487
1	1 (5.0)	9 (7.9)	
2	1 (5.0)	12 (10.5)	
3	7 (35.0)	53 (46.5)	
4	11 (55.0)	40 (35.1)	
**pN stage**			0.385
0	13 (65.0)	76 (66.7)	
1	7 (35.0)	28 (24.6)	
2	0 (0.0)	10 (8.8)	
**AJCC stage**			0.894
1	2 (10.0)	19 (16.7)	
2	11 (55.0)	57 (50.0)	
3	7 (35.0)	38 (33.3)	
Specimen length (cm), median (range)	21.5 (10.0–40.0)	16.0 (6.2–47.0)	0.010
**Margin (cm), median (range)**			
Proximal margin	7.0 (2.0–25.0)	4.0 (1.0–10.5)	<0.001
Distal margin	10.0 (4.5–30.0)	8.0 (2.0–35.0)	0.013
Specimen with an inadequate margin	2 (10.0)	39 (34.2)	0.030
**Lymph node, median (range)**			
Total removed	21.0 (11.0–55.0)	15.5 (2.0–46.0)	0.007
Positive	0.0 (0.0–2.0)	0.0 (0.0–14.0)	0.924

Values are *n* (%) unless otherwise stated. ICA, intracorporeal anastomosis; ECA, extracorporeal anastomosis; VAS, visual analogue scale.

### Pathology results

In patients undergoing ERHC, the maximum tumour diameter, pT stage, pN stage, and AJCC stage were comparable between ICA and ECA. However, the median (range) specimen length (28.3 (13.0–68.0) *versus* 24.0 (4.5–62.0) cm, *P* = 0.042) and distal margin (6.0 (1.5–17.0) *versus* 5.0 (0.5–19.0) cm, *P* = 0.007) were statistically significantly longer in the ICA group. The prevalence of specimens with an inadequate margin in the ICA group was 9.1 per cent, statistically significantly lower than that (20.0 per cent) in the ECA group (*P* = 0.045). The median (range) total number of lymph nodes harvested was statistically significantly greater in patients undergoing ICA than those undergoing ECA (25.0 (10.0–88.0) *versus* 20.0 (2.0–81.0), *P* = 0.036), but there was no statistically significant difference in the number of positive lymph nodes (*Table 1*).

Similar results were observed in patients undergoing TC/ELHC. The ICA group showed a longer specimen length (median (range) of 21.5 (10.0–40.0) *versus* 16.0 (6.2–47.0) cm, *P* = 0.010), a longer proximal margin (median (range) of 7.0 (2.0–25.0) *versus* 4.0 (1.0–10.5) cm, *P* < 0.001), and a longer distal margin (median (range) of 10.0 (4.5–30.0) *versus* 8.0 (2.0–35.0) cm, *P* = 0.013) compared with the ECA group. The prevalence of specimens with an inadequate margin in the ICA group was 10.0 per cent (2/20), statistically significantly lower than that (34.2 per cent, 39/114) in the ECA group (*P* = 0.030). The median (range) total number of lymph nodes harvested was statistically significantly greater in patients undergoing ICA than those undergoing ECA (21.0 (11.0–55.0) *versus* 15.5 (2.0–46.0), *P* = 0.007), but there was no statistically significant difference in the number of positive lymph nodes (*Table 2*).

In addition, the median (range) number of lymph nodes harvested was statistically significantly lower in patients with a specimen with an inadequate margin compared with others (17.0 (2.0–81.0) *versus* 22.0 (3.0–88.0), *P* = 0.007), but there was no statistically significant difference in the number of positive nodes (0.0 (0.0–10.0) *versus* 0.0 (0.0–14.0), *P* = 0.970).

Univariate analysis identified five factors associated with an inadequate margin: BMI, surgical type (TC/ELHC or ERHC), anastomosis method (hand sewn or mechanical), anastomosis approach (ECA or ICA), and pT stage. In multivariate analysis, ECA (OR 2.94 (95 per cent c.i. 1.33 to 6.49), *P* = 0.008) and TC/ELHC (OR 1.75 (95 per cent c.i. 1.03 to 2.96), *P* = 0.038) were independent risk factors for specimens with an inadequate margin (*Table 3*). Besides, ECA and TC/ELHC were still independent risk factors for specimens with an inadequate margin in patients with mechanical anastomosis (*Table S2*) and in patients with side-to-side anastomosis (*Table S3*).

**Table 3:** Univariate and multivariate analysis of risk factors for specimens with an inadequate margin

Risk factors	Univariate analysis		Multivariate analysis	
	OR	*P*	OR (95% c.i.)	*P*
**Sex**				
Male/female	0.85	0.523		
**Age (years)**				
≥60/<60	0.76	0.275		
**BMI (kg/m^2^)**				
≥25/<25	1.63	0.058	1.70 (1.01,2.88)	0.061
**Tumour location**				
Transverse colon/others	1.15	0.593		
**Operative time (min)**				
≥180/<180	0.69	0.258		
**Estimated blood loss (ml)**				
≥100/<100	0.93	0.870		
**Surgical type**				
(TC/ELHC)/ERHC	2.16	0.003	1.75 (1.03,2.96)	0.038
**Anastomosis method**				
Hand sewn/mechanical	2.88	0.022	2.16 (0.84,5.54)	0.108
**Anastomosis approach**				
ECA/ICA	3.37	0.002	2.94 (1.33,6.49)	0.008
**Maximum tumour diameter (cm)**				
<5/≥5	1.32	0.282		
**pT stage**				
T1–2/T3–4	1.93	0.045	1.98 (1.01,3.87)	0.054
**pN stage**				
N1–2/N0	0.98	0.949		

TC, transverse colectomy; ELHC, extended left hemicolectomy; ERHC, extended right hemicolectomy; ECA, extracorporeal anastomosis; ICA, intracorporeal anastomosis.

### Perioperative outcomes

In ERHC, the median (range) operative time was statistically significantly longer in the ICA group compared with the ECA group (150.0 (95.0–260.0) *versus* 140.0 (80.0–329.0) min, *P* = 0.039). However, the median (range) incision length (4.5 (2.5–6.0) *versus* 7.5 (4.0–10.0) cm, *P* < 0.001), recovery of bowel function (2.0 (2.0–3.0) *versus* 3.0 (2.0–4.0) days, *P* = 0.035), and postoperative hospital stay (6.0 (3.0–17.0) *versus* 7.0 (4.0–39.0) days, *P* = 0.042) were all statistically significantly shorter in the ICA group. Besides, the ICA group showed less pain on POD1 (median (range) of 1.5 (1.0–4.0) *versus* 3.5 (1.0–8.0), *P* < 0.001) and less rescue analgesic usage (6 (9.1 per cent) *versus* 35 (20.6 per cent), *P* = 0.036). There were no statistically significant differences in the estimated blood loss or overall incidence of postoperative complications between the two groups (*Table 1*).

Similarly, ICA was associated with a shorter incision length (median (range) of 4.5 (2.5–6.5) *versus* 7.0 (4.0–11.0) cm, *P* < 0.001), an earlier recovery of bowel function (median (range) of 2.0 (2.0–3.0) *versus* 3.0 (2.0–5.0) days, *P* = 0.034), a shorter postoperative hospital stay (median (range) of 6.0 (5.0–15.0) *versus* 7.0 (4.0–19.0) days, *P* = 0.039), less pain on POD1 (median (range) of 2.0 (1.0–4.0) *versus* 4.0 (1.0–8.0), *P* < 0.001), and less rescue analgesic usage (1 (5.0 per cent) *versus* 29 (25.4 per cent), *P* = 0.045) in TC/ELHC. There were no statistically significant differences in the estimated blood loss or overall incidence of postoperative complications between the two groups (*Table 2*).

## Discussion

In this multicentre retrospective comparison of two types of anastomoses after laparoscopic resection of TCC, ICA resulted in longer distal margins in ERHC and longer proximal margins in TC/ELHC. Moreover, a statistically significantly lower prevalence of specimens with an inadequate resection margin and a greater number of lymph nodes harvested were observed in patients undergoing ICA. The authors also found that ECA and TC/ELHC were independent risk factors for specimens with an inadequate margin in laparoscopic resection of TCC.

Recent studies of TCC have mainly focused on perioperative and oncological outcomes of laparoscopic *versus* open radical resection^16,18,24–26^. A handful of studies have found that laparoscopic resection for TCC was associated with better short-term outcomes and similar long-term oncological outcomes^16,18,26^. However, there is massive heterogeneity regarding the surgical procedures included in these studies, in particular in terms of anastomosis. Currently, ICA with a linear stapler and a barbed suture increases the applicability of totally laparoscopic colectomies. Up until now, no study has compared the difference between ICA and ECA in laparoscopic resection for TCC.

Similar to previous studies, the operative time was longer in ICA than ECA in patients undergoing ERHC^9,27–29^. It is well accepted that manual suturing and knotting in the abdominal cavity are responsible for prolonged operative time. However, ICA resulted in decreased postoperative pain and rescue analgesic usage, earlier recovery of bowel function, and shorter length of stay, which is consistent with the results of other studies on laparoscopic colectomy^30,31^. A reasonable explanation may simply be the smaller incision used in ICA (3–5 cm) *versus* that in ECA (6–10 cm). No statistically significant difference in the rate of anastomosis leakage was found, in accordance with the results of a recent meta-analysis of a mixture of randomized and observational studies comparing ICA with ECA after laparoscopic right colectomies^32^.

In terms of pathology, the specimen length was statistically significantly longer in the ICA group, as reported in other studies^13,33^. Only a few studies have compared the difference in resection margins between ICA and ECA groups. In patients who underwent laparoscopic right hemicolectomy with ICA, a longer minimum margin was reported by previous studies (7 *versus* 5 cm, *P* = 0.026^11^; 7.51 *versus* 5.40 cm, *P* = 0.010^13^). Two studies^12,34^ revealed that ICA mainly influenced the distal margin in laparoscopic right hemicolectomy. Similar to these results, ICA resulted in a longer distal margin compared with ECA in patients undergoing ERHC. However, in patients undergoing TC/ELHC, both the proximal and distal margins were statistically significantly longer in ICA.

According to the Japanese Classification of Colorectal, Appendiceal, and Anal Carcinoma^35^, a resection margin of at least 5 cm for CRC is essential in radical colectomy. The 5 cm resection margin was measured *in vivo*. However, CRC specimens are routinely fixed in formalin, which results in shrinkage of the tumour-free margin. A study included 46 CRC specimens and demonstrated that the average shrinkage for the distal resection margin after formalin fixation was 15.3 per cent^22^. Considering the effect of shrinkage, the authors defined a specimen with a minimum margin of less than 4.2 cm as having an inadequate margin. In patients undergoing TC/TLHC, the prevalence of specimens with an inadequate margin was 30.6 per cent (41/134). Of note, this prevalence decreased sharply in ICA (10.0 per cent, 2/20). Similar results were observed in patients undergoing ERHC. In addition, the multivariate analysis demonstrated that ECA was an independent risk factor for specimens with an inadequate margin apart from the TC/ELHC procedure. These findings illustrated that patients with TCC might benefit from ICA with a higher quality of oncological resection margin. A reasonable explanation is that ECA requires greater mobilization and exteriorization of the intestine tube to perform the anastomosis extracorporeally. However, mobilization of the transverse colon can be particularly challenging in comparison with other portions of the colon^36^. When mobilization is insufficient, some surgeons may sacrifice the resection margin rather than extend the incision. In contrast, ICA lowers the need for mobilization of the transverse colon, as the anastomosis is performed intra-abdominally, avoiding the need to exteriorize a thick specimen through a small laparotomy incision^13^. Nonetheless, inadequate margins were found in 10 per cent of specimens in the ICA group, whether the patients underwent ERHC or TC/ELHC. Inadvertent overstretching of the intestines during laparoscopic measurement was a possible reason.

The number of lymph nodes harvested has been found to be a prognostic factor for CRC^37,38^. Similar to the current study, previous studies found that the number of lymph nodes harvested was statistically significantly greater in ICA compared with ECA (19.21 *versus* 15.19, *P* = 0.001)^33^. This can be explained by more mesocolon tissue being removed during this procedure, as attested by the greater length of resected intestines in ICA. Other studies found no statistically significant difference in lymph node harvest between the two groups^11,34,39^. However, these studies, including the current study, were all retrospective studies with limited sample sizes. Notwithstanding, this highlights that the influence of anastomosis on lymph node harvest is still a subject of debate.

This study has several limitations. First, this study was retrospective and the sample size was small. Second, the resection margin measured on the formalin-fixed specimen as a surrogate endpoint was used (instead of the resection margin measured on fresh specimens). Third, this study lacks long-term follow-up and the effect of anastomosis on patient prognosis was not investigated.

Overall, laparoscopic resection of TCC with ICA was associated with superior perioperative outcomes and pathological specimen quality. More adequate resection margins and a greater lymph node yield may be expected in patients undergoing ICA. However, these findings, as well as their prognostic significance, should be verified by further prospective studies.

## Supplementary Material

zrad045_Supplementary_Data

## Data Availability

Raw data are available at https://crc.c-increase.com.
