# Supplementary material for: Pathological and perioperative outcomes of extracorporeal versus intracorporeal anastomosis in laparoscopic transverse colon cancer resection: retrospective multicentre study
Source: BJS Open. 2023 May 10;7(3):zrad045. doi: 10.1093/bjsopen/zrad045 (PMC10170256; doi:10.1093/bjsopen/zrad045)
Supplement: zrad045_Supplementary_Data [file zrad045_supplementary_data.docx]

**Pathological and perioperative and outcomes of extracorporeal versus intracorporeal anastomosis in laparoscopic transverse colon cancer resection: a retrospective multi-center study**

Hao Zhong^1^, Zhenghao Cai^1^, Junyang Lu^2^, Yingchi Yang^3^, Qing Xu^4^, Nan Wang^5^, Liang He^6^, Xiyue Hu^7^, Abraham Fingerhut^8^, Minhua Zheng^1^, Aiguo Lu^1^, Zheng Liu^7^, Yi Xiao^2^, Bo Feng^1^

1 Department of General Surgery, Ruijin Hospital, Shanghai Jiao Tong University School of Medicine, Shanghai, China

2 Department of General Surgery, Peking Union Medical College Hospital, Beijing, China

3 Department of General Surgery, Beijing Friendship Hospital, Capital Medical University, Beijing, China

4 Department of General Surgery, Renji Hospital, Shanghai Jiao Tong University School of Medicine, Shanghai, China

5 Department of General Surgery, Tangdu Hospital, Shanxi, China

6 Department of General Surgery, The first hospital of Jilin University, Jilin, China

7 Department of Colorectal Surgery, Cancer Hospital Chinese Academy of Medical Sciences, Beijing, China

8. Section for Surgical Research, Department of Surgery, Medical University of Graz, Graz, Austria

**Corresponding author:** Bo Feng, Ph.D., Department of General Surgery, Ruijin Hospital, Shanghai Jiao Tong University School of Medicine, No.197, Ruijin Er Road, Shanghai 200025, China. Tel: +86-21-64370045. E-mail: fb11427@rjh.com.cn or Yi Xiao, Ph.D., Department of General Surgery, Peking Union Medical College Hospital, No.1 Shuaifuyuan, Wangfujing, Dongcheng District, Beijing 100730, China. E-mail: xiaoy@pumch.cn or Zheng Liu, Ph.D., Department of Colorectal Surgery, Cancer Hospital Chinese Academy of Medical Sciences, No.17 Panjiayuan Nanli, Chaoyang District, Beijing 100021, China. E-mail: Zheng.liu@cicams.ac.cn.

**Supplementary Materials - Index**

| **Supplementary Figures and Tables** |  |
| --- | --- |
| Table S1. Surgeons’ experience in CRC surgery | *pag. 3* |
| Table S2. Univariate and multivariate analysis of risk factors for specimens with inadequate margin in patients with mechanical anastomosis | *pag. 4* |
| Table S3. Univariate and multivariate analysis of risk factors for specimens with inadequate margin in patients with side-to-side anastomosis | *pag. 5* |

**Supplementary Figures and Tables**

Table S1. Surgeons’ experience in CRC surgery

| Name | Age | Hospital | CRC cases per year |
| --- | --- | --- | --- |
| Bo Feng | 45 | Ruijin Hospital, Shanghai Jiao Tong University School of Medicine | 300 |
| Yi Xiao | 56 | Peking Union Medical College Hospital | 300 |
| Zheng Liu | 42 | Cancer Hospital Chinese Academy of Medical Sciences | 250 |
| Yingchi Yang | 46 | Beijing Friendship Hospital, Capital Medical University | 200 |
| Qing Xu | 50 | Renji Hospital, Shanghai Jiao Tong University School of Medicine | 200 |
| Nan Wang | 43 | Tangdu Hospital | 150 |
| Liang He | 40 | The first hospital of Jilin University | 150 |

CRC: colorectal cancer

Table S2. Univariate and multivariate analysis of risk factors for specimens with inadequate margin in patients with mechanical anastomosis

| Risk factors | Univariate analysis | |  | Multivariate analysis | | |
| --- | --- | --- | --- | --- | --- | --- |
|  | OR | *p* value |  | OR | 95% CI | *p* value |
| Sex |  |  |  |  |  |  |
| Male/Female | 0.82 | 0.454 |  |  |  |  |
| Age (years) |  |  |  |  |  |  |
| ≥60/＜60 | 0.76 | 0.290 |  |  |  |  |
| BMI (kg/m^2^) |  |  |  |  |  |  |
| ≥25/＜25 | 1.50 | 0.135 |  |  |  |  |
| Tumor location |  |  |  |  |  |  |
| Transverse colon/Others | 1.15 | 0.608 |  |  |  |  |
| Operative time (minutes) |  |  |  |  |  |  |
| ≥180/＜180 | 0.66 | 0.235 |  |  |  |  |
| Estimated blood loss (ml) |  |  |  |  |  |  |
| ≥100/＜100 | 1.00 | 1.000 |  |  |  |  |
| Surgical type |  |  |  |  |  |  |
| (TC/ELHC) / ERHC | 2.33 | 0.002 |  | 2.04 | 1.19, 3.50 | 0.010 |
| Type of stapler |  |  |  |  |  |  |
| Circular / linear | 1.43 | 0.188 |  |  |  |  |
| Anastomosis approach |  |  |  |  |  |  |
| ECA/ICA | 3.15 | 0.004 |  | 2.85 | 1.29, 6.28 | 0.009 |
| Maximum tumor diameter (cm) |  |  |  |  |  |  |
| ＜5/ ≥5 | 1.30 | 0.331 |  |  |  |  |
| pT-stage |  |  |  |  |  |  |
| T_1-2_/T_3-4_ | 1.97 | 0.044 |  | 1.81 | 0.91, 3.58 | 0.090 |
| pN-stage |  |  |  |  |  |  |
| N_1-2/_N_0_ | 0.95 | 0.963 |  |  |  |  |

OR: odds ratios; CI: confidence interval.

Table S3. Univariate and multivariate analysis of risk factors for specimens with inadequate margin in patients with side-to-side anastomosis

| Risk factors | Univariate analysis | |  | Multivariate analysis | | |
| --- | --- | --- | --- | --- | --- | --- |
|  | OR | *p* value |  | OR | 95% CI | *p* value |
| Sex |  |  |  |  |  |  |
| Male/Female | 0.67 | 0.241 |  |  |  |  |
| Age (years) |  |  |  |  |  |  |
| ≥60/＜60 | 0.63 | 0.172 |  |  |  |  |
| BMI (kg/m^2^) |  |  |  |  |  |  |
| ≥25/＜25 | 1.89 | 0.069 |  | 1.86 | 0.91,3.80 | 0.088 |
| Tumor location |  |  |  |  |  |  |
| Transverse colon/Others | 1.41 | 0.407 |  |  |  |  |
| Operative time (minutes) |  |  |  |  |  |  |
| ≥180/＜180 | 0.68 | 0.384 |  |  |  |  |
| Estimated blood loss (ml) |  |  |  |  |  |  |
| ≥100/＜100 | 1.10 | 0.884 |  |  |  |  |
| Surgical type |  |  |  |  |  |  |
| (TC/ELHC) / ERHC | 2.91 | 0.002 |  | 2.44 | 1.20, 4.95 | 0.014 |
| Linear anastomosis |  |  |  |  |  |  |
| Isoperistaltic / antiperistaltic | 1.10 | 0.786 |  |  |  |  |
| Anastomosis approach |  |  |  |  |  |  |
| ECA/ICA | 3.13 | 0.007 |  | 2.72 | 1.17, 6.34 | 0.020 |
| Maximum tumor diameter (cm) |  |  |  |  |  |  |
| ＜5/ ≥5 | 1.22 | 0.567 |  |  |  |  |
| pT-stage |  |  |  |  |  |  |
| T_1-2_/T_3-4_ | 1.52 | 0.329 |  |  |  |  |
| pN-stage |  |  |  |  |  |  |
| N_1-2/_N_0_ | 1.15 | 0.686 |  |  |  |  |

OR: odds ratios; CI: confidence interval.
